# Base-Pairing Versatility Determines Wobble Sites in tRNA Anticodons of Vertebrate Mitogenomes

**DOI:** 10.1371/journal.pone.0036605

**Published:** 2012-05-09

**Authors:** Miguel M. Fonseca, Sara Rocha, David Posada

**Affiliations:** 1 CIBIO, Research Center in Biodiversity and Genetic Resources, University of Porto, Porto, Portugal; 2 Department of Biochemistry, Genetics and Immunology, University of Vigo, Vigo, Spain; Newcastle University, United Kingdom

## Abstract

**Background:**

Vertebrate mitochondrial genomes typically have one transfer RNA (tRNA) for each synonymous codon family. This limited anticodon repertoire implies that each tRNA anticodon needs to wobble (establish a non-Watson-Crick base pairing between two nucleotides in RNA molecules) to recognize one or more synonymous codons. Different hypotheses have been proposed to explain the factors that determine the nucleotide composition of wobble sites in vertebrate mitochondrial tRNA anticodons. Until now, the two major postulates – the “codon-anticodon adaptation hypothesis” and the “wobble versatility hypothesis” – have not been formally tested in vertebrate mitochondria because both make the same predictions regarding the composition of anticodon wobble sites. The same is true for the more recent “wobble cost hypothesis”.

**Principal Findings:**

In this study we have analyzed the occurrence of synonymous codons and tRNA anticodon wobble sites in 1553 complete vertebrate mitochondrial genomes, focusing on three fish species with mtDNA codon usage bias reversal (L-strand is GT-rich). These mitogenomes constitute an excellent opportunity to study the evolution of the wobble nucleotide composition of tRNA anticodons because due to the reversal the predictions for the anticodon wobble sites differ between the existing hypotheses. We observed that none of the wobble sites of tRNA anticodons in these unusual mitochondrial genomes coevolved to match the new overall codon usage bias, suggesting that nucleotides at the wobble sites of tRNA anticodons in vertebrate mitochondrial genomes are determined by wobble versatility.

**Conclusions/Significance:**

Our results suggest that, at wobble sites of tRNA anticodons in vertebrate mitogenomes, selection favors the most versatile nucleotide in terms of wobble base-pairing stability and that wobble site composition is not influenced by codon usage. These results are in agreement with the “wobble versatility hypothesis”.

## Introduction

Twelve of all 13 protein-coding genes encoded on the vertebrate mitogenomes are collinear with the AC-rich light-strand, while ND6 is the only protein-coding gene located in the opposite strand (heavy-strand) [1]. Overall codon usage is therefore mostly determined by these 12 protein-coding genes, which contain a high frequency of AC-ending codons. The vertebrate mtDNA genetic code has 60 amino acid codons (and two termination codons [2]), but typically have only one type of transfer RNA molecule (tRNA) for each amino acid codon family. A codon family consists of all synonymous codons, which differ only in their third codon position but code the same amino acid. This fact implies that each tRNA anticodon must wobble with one or more nucleotides to recognize all codons in a synonymous codon family [3]. A wobble base-pair is a non-Watson-Crick base pairing between two nucleotides in RNA molecules and hence it is less stable than a Watson-Crick base pairing.

Several studies have focused on the evolution of tRNA anticodons and codon usage in different organisms and organelles (e.g., [4]–[9]). Regarding vertebrate mitogenomes, two main contrasting hypotheses have been proposed to explain which factors determine the wobble nucleotide of tRNA anticodons, the “codon-anticodon adaptation hypothesis” [5] and the “wobble versatility hypothesis” [10]. The codon-anticodon adaptation hypothesis, or CAAH, states that codon usage determines the nucleotide at the wobble site of the tRNA anticodon, implying that the wobble site should co-evolve with codon usage and match the most frequent codon in a given synonymous family. This hypothesis was originally invoked to explain the correlation between codon abundance and anticodon composition in vertebrate mitogenomes [5]. Amino acid codons can be divided in NNN, NNY and NNR synonymous codon families (where N stands for any of the four nucleotides, Y stands for either C or U and R stands for either A or G). In animal mitochondrial genomes NNY codons end mostly with C, while NNR and NNN codons end mainly with A. Therefore, the CAHH prediction for the wobble site of the tRNA anticodons is wobble G for the NNY codon family, and wobble U for NNR and NNN codon families. In contrast, the wobble versatility hypothesis, or WVH, proposes that the composition of wobble sites is independent of codon usage and is selected to maximize its versatility to pair with all members of a synonymous codon family, i.e., the wobble site should be occupied by the most versatile nucleotide in wobble-pairing. The predictions for the anticodon sites according to the WVH are G at wobble sites for NNY codon families, because G can wobble with C and U; and U for NNR and NNN codon families, since U is the base that can pair most effectively with all 4 third-position bases [10], [11]. Consequently, both the CAAH and WVH hypotheses make the same predictions for the anticodon wobble sites and both are compatible with the nucleotide composition of vertebrate mitogenomes [6]. However, there is one exception, the tRNA-Met, for which the wobble composition is not in agreement with either of the hypotheses [5], [10]. The anticodon of tRNA-Met is the only one having C at the wobble site instead of U. Consequently, the tRNA-Met anticodon forms a Watson-Crick match with the AUG codon instead of the AUA codon, despite the fact that the latter is much more abundant. The codon AUG not only codes for methionine, but is also known to be the most frequent and efficient initiation codon [12]–[15]. The anticodon of tRNA-Met matching AUG favors translation initiation rates and not translation elongation efficiency. This conflict between translation initiation and elongation was proposed to explain the usage of CAU anticodon only for tRNA-Met of vertebrate mitogenomes, giving rise to the “translational conflict hypothesis” (TCH) [16].

More recently, it has been proposed that anticodon wobble sites of tRNA should be occupied by a nucleotide that minimizes reduction in decoding efficiency and accuracy, the so-called “wobble cost hypothesis” (WCH) [17]. The WCH can be seen as an integration of CAAH and WVH that explains wobble nucleotide choice by the cost associated to each wobble base-pair in each genome. These costs will depend on codon usage, which is the main difference relative to the WVH. The predictions of WCH and WVH are identical unless extreme codon usage alters the relative costs of wobble pairings – which happens only when the frequency of the third codon base that pairs at no cost with the wobble site is very low or even null. The WCH was initially tested in a dataset composed by 36 fungal mitogenomes: Xia [17] found two examples in fungal mitogenomes where the wobble site changed to a less versatile nucleotide (G -> A) in two NNY codon families with very low frequency of C at the third codon position (Asn - AAY and Ser - AGY codon families). Indeed, Xia suggested that a less versatile wobble A at the tRNA anticodon was advantageous over a wobble G because the cost of having a wobble G for these codon families with very low frequency of the complementary third codon position nucleotide (codons AAU (Asn) and AGU (Ser)) was higher than having a less versatile wobble A complementary to the most frequent third codon position nucleotide (codons AAC (Asn) and AGC (Ser)). So far, WCH has not been tested in vertebrate mitogenomes.

Vertebrate mitogenomes typically encode the same set of 22 tRNAs. Presumably, all protein-coding genes on the mitochondrial genome are essential genes and have expressions levels that do not vary greatly. Overall codon usage shows some variation, but typically reflects the direction of the strand-specific mutation bias (AC-ending codons). Thus, in vertebrate mitogenomes, the gene expression levels, the number and type of tRNAs and codon usage make these genomes unsuitable to study coevolution between tRNAs and codon usage because these genomes are basically at equilibrium. However, some vertebrate mitogenomes have suffered a codon usage reversal [18]), providing a unique opportunity to study coevolution between tRNAs and codon usage. In these genomes, the fact that codon usage changed from AC-rich to GU-rich allow us to investigate on some key questions respect wobble sites evolution: did the wobble site in tRNA anticodons also change to match the new most frequent codons (supporting CAAH or WCH) or not (in agreement with WVH)? Are there any evidences at the codon usage level suggesting different costs between the two kinds of U:G wobble pairs proposed by WCH? Here, we have analyzed the occurrence of synonymous codons and tRNA anticodon wobble sites of all 1553 available complete vertebrate mitogenomes, with an emphasis on the three fish mitogenomes with independent codon bias reversal. Our analyses provide further insights into the influence of anticodon-codon interactions on codon usage and allow us to contrast the different hypotheses proposed to explain wobble site composition in tRNA anticodons.

## Results

**Figure 1:**
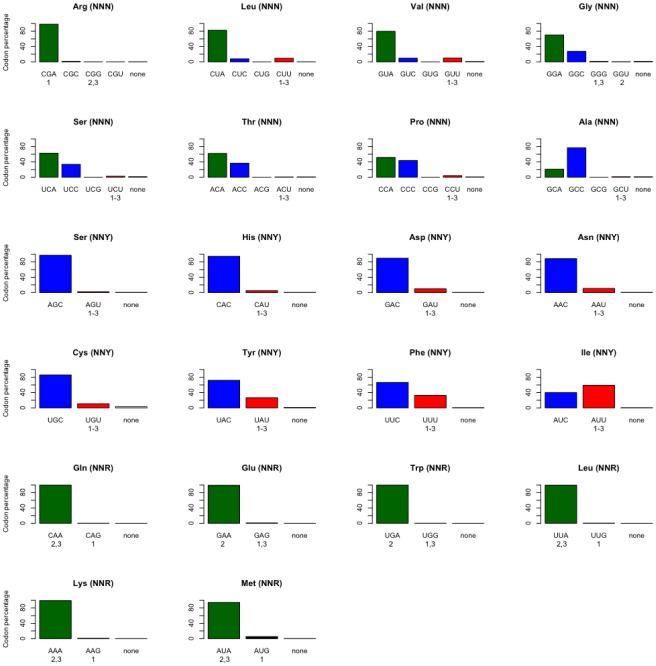
Most abundant codons found in each amino acid of vertebrate mitogenomes. If for a given amino acid there was more than one most abundant codon in a mitogenome, then we considered that there was no most abundant codon (“none”). The mitogenomes of the three fish species with codon usage reversal are indicated in numbers below their respective exhibited codon: 1-*Albula glossodonta* (NC_005800), 2-*Bathygadus antrodes* (NC_008222), and 3-*Tetrabrachium occelatum* (NC_013879).

Our analyses indicate that vertebrate mitogenomes have A and C as the most abundant nucleotides at the third codon positions, which is consistent with the overall compositional bias found in the light-strand [1]. In NNN codon families (each mitogenome has 8 NNN codon families), 99.4% are AC-rich at the third codon position (figure 1). Similarly, 98.7% of the NNR codon families (each mitogenome has 6 NNR codon families) have A as the most abundant third codon position nucleotide rather than G. For the NNY codon families (each mitogenome has 8 NNY codon families), the frequency of C drops to 80.3%. However, not all codon families follow the exact same pattern. In NNR codon families, A-ending codons are clearly most abundant, but in NNN and NNY codon families there is more variability. For example, C-ending codons have a frequency of 76.8% for the amino acid Alanine (CGN codon families, tRNA molecule with wobble U), but T-ending codons appear in 59.2% of mitogenomes for the amino acid Isoleucine (AUY codon family, tRNA molecule with wobble G). Remarkably, there are three fish mitogenomes that show overall codon usage reversal, i.e., they are rich in GU-ending codons (*Albula glossodonta*, *Bathygadus antrodes* and *Tetrabrachium ocellatum*) (figure 1). In these genomes, a codon usage reversal is clear in NNN and NNY codon families but not in NNR codon families in which the reversal is only pronounced in the *A. glossodonta* mitogenome.

In our analysis, virtually all tRNAs have wobble G or U at the anticodons, except tRNA-Met that presents wobble C. This is in agreement with what was previously described for vertebrate mitogenomes [5], [10], [16]. There are a few exceptions that most likely are sequencing errors (table 1 and ref. [10]), as most imply a wobble nucleotide that would not allow for the decoding of all codons for the given synonymous codon family. Additionally, there is no evident codon-bias in the direction of the nucleotide matching the new wobble position in none of these exceptions, and thus one would have to assume a highly ineffective translation/elongation processes if these wobble sites were to be true. Most importantly, the three mitogenomes with codon usage reversal presented the same wobble nucleotides at the anticodons as all the remaining vertebrate mitogenomes and hence none of the wobble sites of tRNAs anticodons coevolved with the codon usage reversal.

**Table 1:** Exceptions found for the wobble nucleotide of tRNA anticodons.

tRNA	Codon Family	Wobble		Codon Usage (%) b				Codon Usage Across Genomes [MEAN (MIN-MAX)] % c				RefSeq d	Species
		Expected a	Observed	A	C	G	T	A	C	G	T		
Ala	NNN	U	C	30.6	48.4	3.3	17.7	34,1 (15,9–55,2)	42,7 (16,3–65,9)	3,7 (0–14,4)	19,6 (7,0–46,3)	NC_004381	*Chaunax* *abei*
			C	28.9	48.4	3.8	18.9					NC_004382	*Chaunax tosaensis*
			C	29.4	48.6	3.9	18.1					NC_013883	*Chaunax* *pictus*
Arg	NNN	U	C	64.8	18.9	9.5	6.8	58,6 (19,8–84,0)	19,8 (1,4–55,0)	9,5 (0–38,7)	12,2 (0–34,2)	NC_010199	*Odontobutis* *platycephala*
			C	70.0	12.9	1.4	15.7					NC_010970	*Cyclemys* *atripons*
Leu	NNN	U	A	21.7	33.2	18.0	27.1	45,7 (14,0–71,5)	23,1 (4,79–44,5)	8,6 (1,3–24,8)	22,6 (5,5–51,3)	NC_006917	*Jenkinsia* *lamprotaenia*
	NNN	U	C	32.2	22.0	11.7	34.1					NC_006131	*Acanthogobius* *hasta*
Pro	NNN	U	G	30.8	42.4	5.1	21.7	40,1 (7,5–83,4)	34,8 (3,7–70,6)	4,3 (0–16,4)	20,7 (4,3–52,1)	NC_002504	*Lama* *pacos*
			C	55.1	29.9	4.7	10.3					NC_014175	*Acanthosaura* *armata*
Val	NNN	U	C, A	26.7	30.1	14.4	28.8	40,2 (14,1–64,8)	23,0 (5,2–42,8)	11,1 (1,2–28,9)	25,7 (10,1–44,4)	NC_004409	*Lycodes* *toyamensis*
			A	55.0	20.2	3.1	21.7					NC_009421	*Chlamydosaurus* *kingii*
			C	50.3	13.5	10.3	25.9					NC_011218	*Canis lupus* *laniger*
Asp	NNY	G	A		77.9		22.1		66,3 (20,6–91,0)		33,7 (8,9–79,4)	NC_015232	*Cyanoptila* *cyanomelana*
Phe	NNY	G	A		45.4		54.6		54,3 (12,9–85,8)		45,7 (14,2–87,1)	NC_007179	*Cervus nippon* *yakushimae*

a Wobble nucleotide in all remaining vertebrate mitogenomes for the given tRNA;

b Codon usage measured in the RefSeq mitogenome for the specified amino acid synonymous codon family (first column);

c Codon usage % values (mean, lowest, highest) measured across all vertebrate mitogenomes for the specified amino acid synonymous codon family (first column);

d NCBI accession number.

## Discussion

Our survey shows that wobble sites of tRNAs anticodons do not always match the most frequent third codon position for a given codon family, in disagreement with the predictions made by the CAAH. Moreover, in the three mitogenomes with codon usage reversal the wobble sites did not coevolve accordingly. If codon usage drives the evolution of the wobble sites of tRNA anticodons, as suggested by CAAH, then selection would favor a compositional change at the wobble site of tRNAs anticodons in these three atypical mitogenomes in order to match the new most frequent codons [5]. In the NNN codon families the three fish mitogenomes have mostly GU-ending codons but the corresponding tRNA anticodons have still wobble U. Likewise, for the NNY codon families, the wobble site is also U even though most codons end in U in these three fish mitogenomes. Hence, in vertebrate mitogenomes the wobble position of tRNAs is fixed to be U for NNN/NNR codon families (except tRNA-Met with wobble C) and G for NNY codon families.

It may be argued that there wasn’t enough evolutionary time for the tRNAs to change its wobble position after codon usage reversal and therefore our results do not necessarily support WVH. We do not agree with this argument: the strong codon usage reversal found in these genomes suggests that there has indeed been enough time to change overall nucleotide composition along the mitogenome. The fact that all wobble sites in tRNA anticodons are strongly conserved in genomes with overall codon usage reversal is concordant with the predictions of the WVH [10]. Most codon families from mitogenomes of marine bivalves, hemichordata and fungus support the WVH [4], [6], [10], [19].

**Figure 2:**
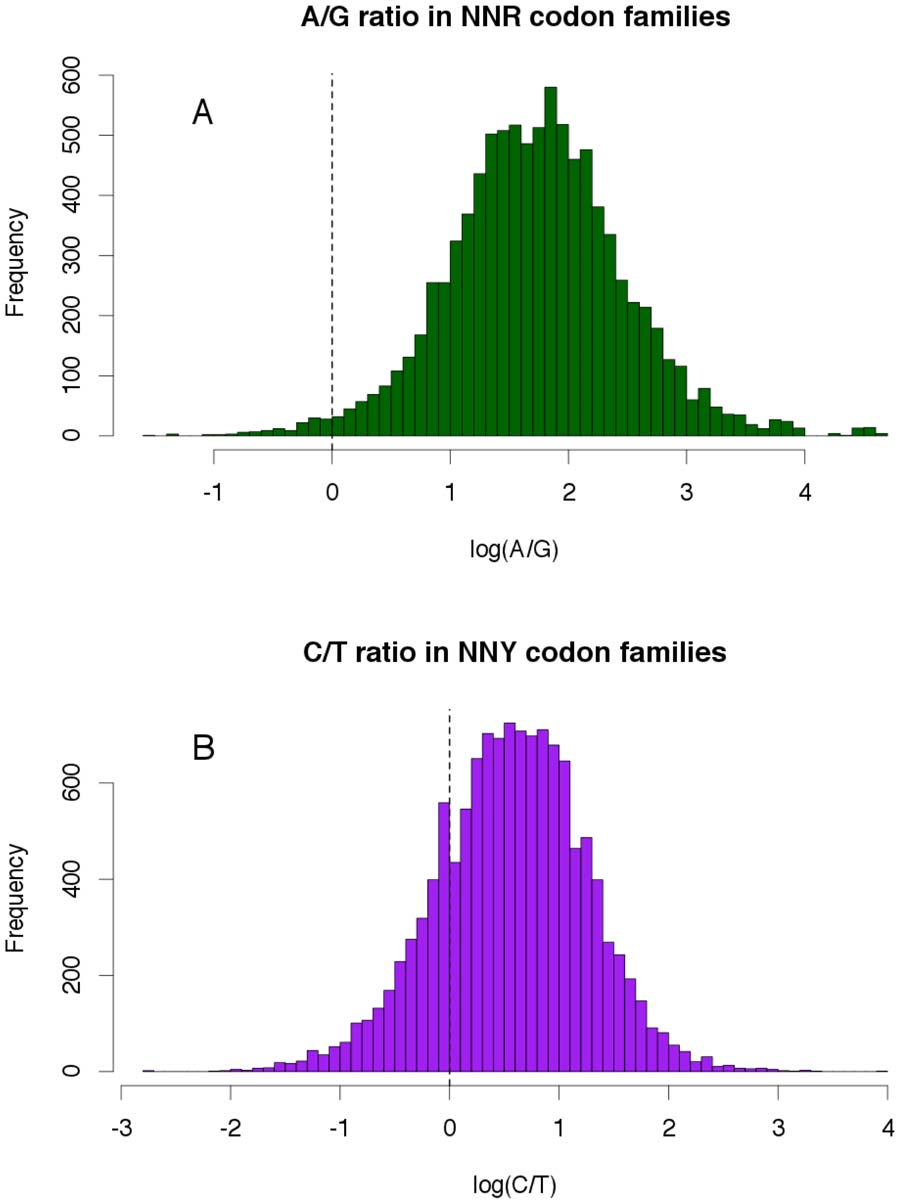
Distribution of the A/G and C/U rations in vertebrate mitogenomes. (A) Histogram with the distribution of log_10_(A/G) ration in NNR codon families. (B) Histogram with the distribution of log_10_(C/U) ration in NNR codon families. Dashed line indicates log_10_(ratio) = 0. Negative log_10_(ratio) values mean that for a given codon family in a given genome G is more frequent than A (Figure 2A) and U are more frequent than C (Figure 2B).

On the other hand, the WCH predicts that the wobble site of tRNAs anticodons may change if the cost of maintaining the original wobble nucleotide becomes a selective disadvantage for the organism. This scenario may happen when the frequency of the third codon nucleotide of a given codon family, complementary to the wobble site of the tRNA anticodon of that same codon family, is very low or even null. A wobble change from G to A was observed in two fungal mitogenomes in a NNY codon family for which the frequency of non-complementary third codon position nucleotide was more than ten times as frequent as the complementary one [17]. The observed C/U ratios were 0.0870 (*Penicillium marneffei*) and 0.0083 (*Pichia canadensis*), while the same ratio calculated for the remaining fungal mitogenomes that maintained the wobble nucleotide was 0.1950 [17]. In our survey, the three fish mitogenomes with the codon usage reversal also presented, for some codon families, smaller C/U and A/G ratios (C/U = 0.066 A/G = 0.208; figure 2) than the smallest C/U and A/G ratios from the remaining vertebrate mitogenomes (C/U>0.130 and A/G>0.260; figure 2). Either these ratios are not small enough to promote a wobble change to the nucleotide complementary to the most frequent third codon position i.e. the translational cost of having a wobble site matching the most frequent codon is still higher than maintaining the original wobble site that does not matches the most frequent codon, or the WCH does not apply to vertebrate mitogenomes (with the translational system being able to function well based on relaxed wobble pairing rules and only with one tRNA for each codon family [10]).

In summary, our survey indicates that in vertebrate mitogenomes the wobble base of tRNAs anticodons is conserved and determined by its pairing-versatility, as proposed by the wobble versatility hypothesis. Overall, it seems that intrinsic characteristics that govern nucleotide pairing are more important to tRNA anticodon evolution than overall mutational pressure, and that selective factors play an important role in determining these positions.

## Materials and Methods

We analyzed all complete vertebrate mitogenomes publicly available in NCBI until 13^th^ May 2011, totaling 1553 mitogenomes. Annotations from the original Genbank files were checked and corrected if necessary before further analyses. Ten mitogenomes were not analyzed in terms of tRNA anticodons because their GenBank records contained misannotations. Nucleotide bias was summarized as GC and AT skews: AT skew = (A – T)/(A+T), GC skew = (G – C)/(G+C) [20]. Codon usage for all protein-coding genes was calculated using in-house Perl scripts. Transfer RNA genes were identified using ARWEN [21] and further screened for possible false positives using copy number and structural information: tRNA conservation at primary and secondary structure, tRNA location and coding direction. All graphs and statistics were implemented using R 2.12.0 [22].
